# The Nest Architecture of Three Species of North Florida *Aphaenogaster* Ants

**DOI:** 10.1673/031.011.10501

**Published:** 2011-08-22

**Authors:** Walter R. Tschinkel

**Affiliations:** Department of Biological Science, Florida State University, Tallahassee, FL 32306-4370

**Keywords:** colony size, size-free shape, nest area, excavation, subterranean nests, worker number, *Aphaenogaster floridan Aphaenogaster treatae*, *Aphaenogaster ashmeadi*

## Abstract

The architecture of the subterranean nests of *Aphaenogaster floridana* Smith (Hymenoptera: Formicidae), *A. treatae* Forel and *A. ashmeadi* (Emery), was studied from plaster, wax, or metal casts. After structural features were quantified from digital images, the entombed ants were retrieved from the plaster by dissolution or wax casts by melting and counted. Nests of all three species were rather simple, small and vertical, with horizontal chambers connected by vertical shafts. Shafts descending to lower chambers tended to arise from chamber edges, whereas those connecting to a chamber above tended to arise from chamber centers. *A. floridana* had the largest nests and colonies, and multiple shafts commonly connected upper chambers, a feature lacking in the other two species. In *A. floridana* nests a higher proportion of chamber area and greater spacing between chambers occurred in the deeper parts of the nest, regardless of nest size. The other two species showed no vertical differentiation of any size-free measure at any nest size. In all three species, nest size increased more slowly than the worker population, so crowding was greater in large colonies than in small, in contrast to the situation in three other ant species for which data were available. An appendix with stereo images of all casts is provided.

## Introduction

When applied to ant colonies, the superorganism metaphor suggests that the subterranean nest constructed by the ants is a functional part of the superorganism and is the product of natural selection. Just as morphological differences among nonsocial organisms reflect both historical and functional differences, so should differences in the nest architecture of ants reflect historical and functional differences. Nest architecture is as much a part of the life cycle as colony size, season of breeding, and reproductive output.

The relationship between particular architectural elements and colony function is mostly unknown, but logically the study of nest architecture can potentially lead to important understanding of how ant colonies work. Unfortunately, the study of subterranean ant nest architecture is in its infancy. A modest literature of mostly descriptive studies is beginning to outline the range of architectural variation within and among species (reviewed by Tschinkel 2004), but the functional meaning of this variation has rarely been addressed. Most reports provide only verbal descriptions or simple drawings based on excavations, and very few included a census of the colony or quantitative details of the architecture, but more recent studies (Tschinkel 1987, 1999, 2003, 2004, 2005; Mikheyev and Tschinkel 2004; Cerquera and Tschinkel 2010) provide quantitative and qualitative analysis based on substantial sample sizes. In addition, the architecture of the nests of the fungus-gardening ants has received more attention than most other groups (Jonkman 1980a, 1980b; Mueller and Wcislo 1998; Moreira et al. 2004a, 2004b; Solomon et al. 2004; Fernández-Marín et al. 2005; Klingenberg et al. 2006; Verza et al. 2007; Rabeling et al. 2007).

Nevertheless, ants clearly excavate species-typical subterranean nests, a conclusion strengthened by more recent work (Tschinkel 1987, 1999, 2003, 2004, 2005; Mikheyev and Tschinkel 2004; Cerquera and Tschinkel 2010; Plaza and Tinaut 1989; Ruano and Tinaut 1993; Moreira et al. 2004a, 2004b; Diehl-Fleig and Diehl 2007; Forti et al. 2007; Vieira et al. 2007). Despite an enormous range of size, a large proportion of ant nests are composed of two basic elements that include more or less vertical shafts connecting horizontal chambers (Tschinkel 2003). The architectural variation among species is largely the result of variation in the form, spacing, and size of these elements. Nests with similar architecture can vary from a few centimeters deep to 4 m or more (Tschinkel 2003).

Because nest excavation is a group activity, the manner in which the architecture results from self-organized behavior has stimulated experimental and modeling analysis of ant tunneling activity (Buhl et al. 2006; Rasse and Deneubourg 2001). Gas gradients in ant nests have been modeled because they have been suggested as templates for nest construction (Cox and Blanchard 2000; Tschinkel 2004). New study methods include x-ray computed tomography, which has been applied to the study of the growth of small Argentine ant nests in the laboratory (Halley et al. 2005). Trace fossils interpreted as having been constructed by ants have also drawn considerable interest (for a review, see Hasiotis 2003).

However, as in any young field, it is first necessary to describe, in quantitative terms the structure and range of variation of the nests of a variety of ant species, as well as the distribution of the ants within these structures. This paper provides a description of the nest architecture and its variation for three species belonging to the genus *Aphaenogaster* (Hymenoptera: Formicidae), and together with several previous papers (Tschinkel 1987, 1999, 2003, 2004, 2005; Mikheyev and Tschinkel 2003; Cerquera and Tschinkel 2010), contributes to the beginnings of a systematic and comparative study of ant nest architecture for its own sake. Although the Tallahassee area is home to eight species of *Aphaenogaster*, only three of these build subterranean nests that are common enough to study.

## Materials and Methods

### Study site

Study populations were located at three different sites in the Apalachicola National Forest, two in the sandhills region and one in the flatwoods. The sandhills sites were occupied primarily by *Aphaenogaster floridana* Smith and *A. ashmeadi* (Emory), whereas the flatwood site was home mostly to *A. treatae* Forel with less representation by *A. floridana. A. ashmeadi* was absent from the flatwoods site.

### Nest casting

Details of making casts of subterranean ant nests can be found in Tschinkel (2010). Briefly, a thin slurry of dental plaster in water was poured into the nest entrance and allowed to set for about an hour, and the hardened cast excavated. Casts always broke, and after drying the laboratory, the pieces were assembled with 5-min epoxy cement. Toward the end of the study, several casts were made with molten paraffin wax and several with molten aluminum (Tschinkel 2010). When aluminum is used nests deeper than 1 m must be cast in stages because the aluminum freezes before filling the nest. After removal of the first stage, the continuation of the nest is exposed and aluminum is poured again. Several repetitions of this process may be necessary to produce a complete cast. The stages are welded together later. Plaster casts of deep nests must also be cast in stages. None of the *Aphaenogaster* nests in this study were deep enough to require casting in stages.

### Data collection

Casts were digitally photographed with a scale, and measurements of dimensions and spacing were made from these photographs. After the casts were photographed, they were broken into chambers and shafts, and the pieces laid flat on a black background with a scale and photographed from above. Chamber area and perimeter measurements were made from these digital images.

### Recovering the ants for census

Ants can be recovered from plaster and wax castes. In the case of plaster castes, the broken cast pieces were tied into fine mesh bags and placed in slowly running hot water. In the course of about a month, all the plaster dissolved, and the ants remained in the bags (although in pieces). The census was literally based on counts of heads. Casts made with wax were melted in a beaker and the ants recovered intact for census and study.

The heads of gynes were easily recognized by their size and the presence of ocelli, but the queen mother of the colony could not be distinguished from female alates. In many cases with multiple gyne heads, mesonota with wings were also present, suggesting that most of the gynes had been winged.

### Head-width measurements

**Figure 1.**
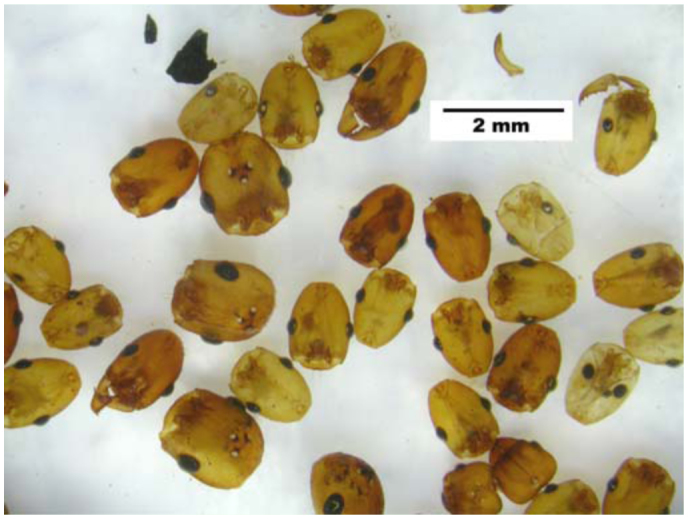
An example of the heads *of Aphaenogaster floridana,* along with the scale used for their measurement. These heads were retrieved after the plaster nest cast was dissolved in hot water. Note that some heads are clearly teneral. Note also that one of the heads is not *A.* *floridana.* High quality figures are available online.

The heads recovered from the casts were placed within outlined rectangles representing the field of view of a dissecting microscope. The card with the rectangles was covered with double-stick tape, so that the heads were held in place. These fields were photographed with a scale (Figure 1), and the head widths estimated from the digital images.

### Statistical analysis

Data were analyzed according to standard procedures such as analysis of variance and regression provided by Statistica 6 (Statsoft, Inc.). When necessary, data were log transformed for stabilization of the variance. Reviewers often object to regressing log x/y against log y, that is, regressing a ratio on the denominator of the ratio, usually suggesting instead a regression of log x on log y followed by a comparison of the slope to 1.0 (isometry), but Mosimann and James (1979) point out that the regression of the log x/y on log y is equivalent to testing whether the slope of the x-y regression is different from 1.0 (isometry)—significant differences show up as positive or negative slopes, and isometry shows up as a slope of zero. The meaning of the ratio plots for shape analysis is more easily seen, because changes of shape in relation to the magnitude of y are seen as nonzero slopes. I have followed Mosimann and James' (1979) suggestions for such analyses.

## Results

**Figure 2.**
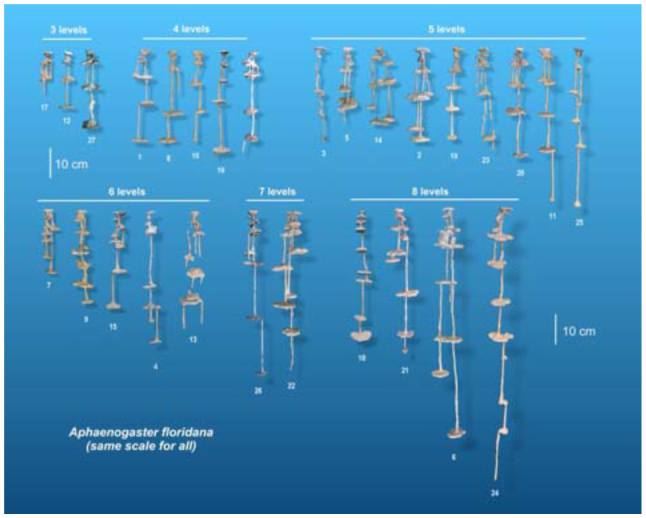
Nest casts of *Aphaenogaster floridana,* all to the same scale and grouped by the number of “levels.” The variation in maximum nest depth within each group suggests that spacing between chambers or levels contributes to maximum depth independently. The number under each cast is the number of the Appendix image in which a larger stereo image of the cast can be found. High quality figures are available online.

Figures 2, 10 and 14 show the nests of each species to the same scale to allow comparison of nests of different sizes. A higher-resolution, stereo image of each of these casts can be found in Appendices 1 to 27 for *Aphaenogaster floridana*, Appendices 28 to 39 for *A. treatae*, and Appendices 40 to 52 for *A. ashmeadi.* The number under each cast is an identification number of each cast, and for convenience is identical to the appendix number. By proper ocular techniques or the use of a stereo viewer, the stereo images in the Appendices can be seen in three dimensions. All three species build nests of small to moderate size, with rather simple architecture but consistent differences among the species. The qualitative and quantitative features of each species are discussed below, followed by a comparison of the species.

### Architectural features of *A. floridana*

**Figure 3.**
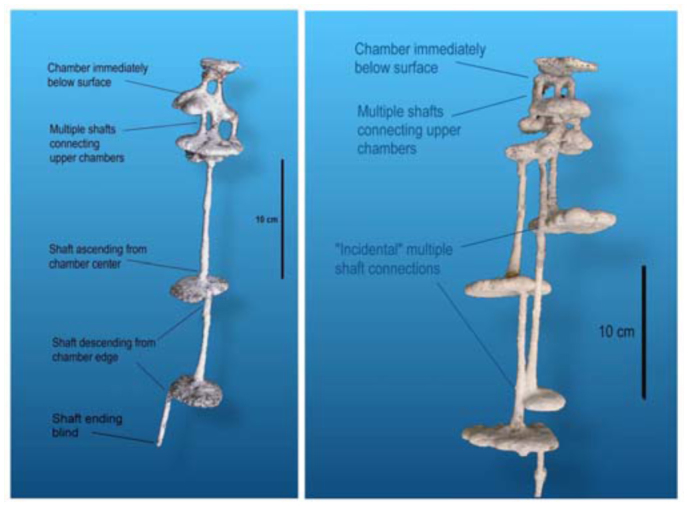
Consistent architectural features of the nests of *Aphaenogaster floridana*, illustrated with two examples.High quality figures are available online.

Nests of *A. floridana* show several conspicuous, consistent structural features (Figures 2, 3). First all nests are highly vertical and “linear”; that is, little lateral spread occurs, and all chambers lie directly below the nest entrance. Second, all have a small chamber immediately below the surface. Slightly enlarging the nest entrance in living nests often provides a view of the floor of this upper chamber. Another consistent feature is that, in almost all nests, chambers in the upper quarter (or less) are connected to each other by two or three shafts, whereas most of the lower connections are single shafts. Lower parts of the nest may include multiple shafts, but they connect separate pairs of chambers (for example, Figure 2, nests 2, 4, 5, 6, 7, 14). When these deeper multiple shafts connect chambers, one of them often seems to connect incidentally on the way to a deeper chamber (for example, Figure 2, nests 2, 6, 7, 9, 15, 21, 22, 26).

**Table 1.**
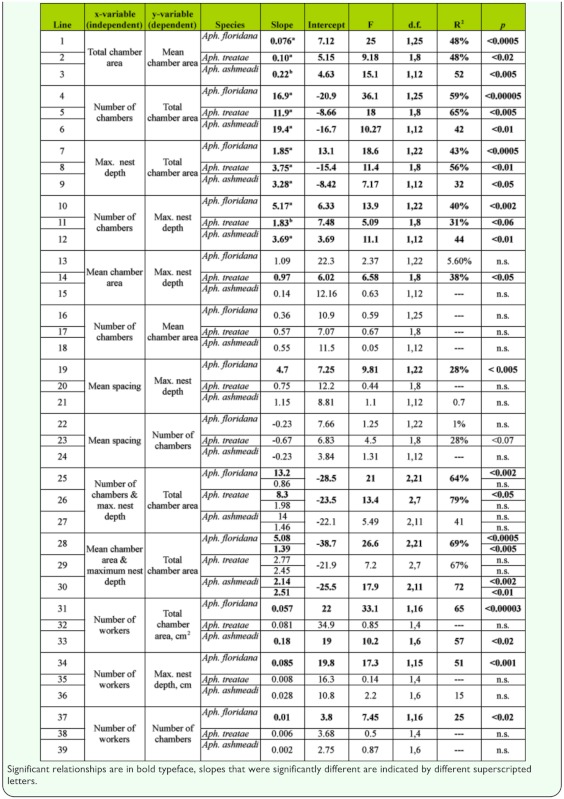
Regression of nest size variables. In these regressions, the species were regressed singly.

**Figure 4.**
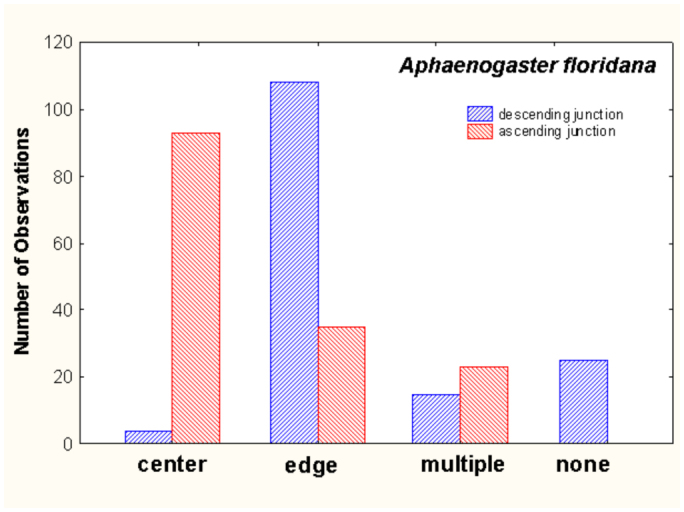
*Aphaenogaster floridana* shafts connecting to a chamber above usually arose from the center of a chamber, whereas those descending to a chamber below arose from the chamber's edge. Multiple connections between sequential chambers were mostly found in the upper quarter (or less) of the nest. High quality figures are available online.

Another consistent feature is that shafts descending to chambers below tend to begin from the edge of the chamber, whereas those connecting to the chamber above usually arise from the center of the chamber (Figure 4). This pattern is especially strong in the bottom half of the nest (for example, Figure 2, nests 1, 8, 10, 13, 15, 22, 26). The pattern may arise as a result of episodic chamber excavation in which, after a period of inactivity, the ants initiate a vertical shaft at the chamber's edge and, at the bottom of the new shaft, form a circular chamber by excavating outward equally in all directions, so that the shaft descending from the chamber above connects to the center of the new chamber. Several nests also included shafts that did not end in chambers (for example, Figure 2, nests 5, 13, 22, 24), suggesting the initial phase of this process.

**Figure 5.**
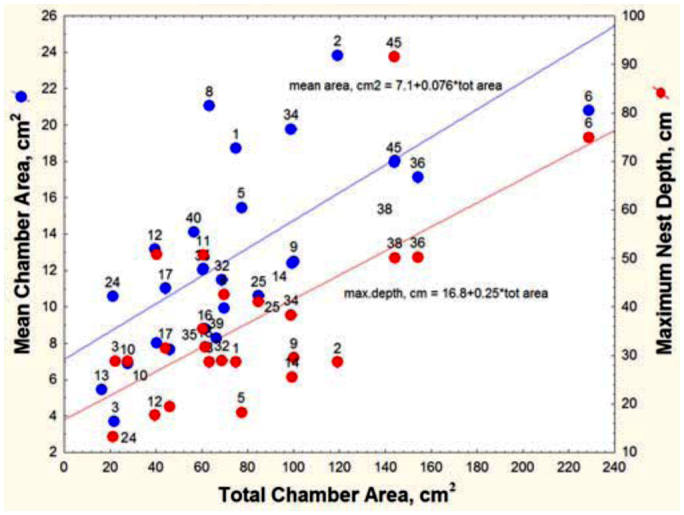
As nest size (total chamber area) increased, the mean chamber increased in area, and the nest deepened. Mean chamber size and maximum depth both increased 4- to 5-fold over the range of total size. The number next to each point refers to the nest number in Figure 2. High quality figures are available online.

**Figure 6.**
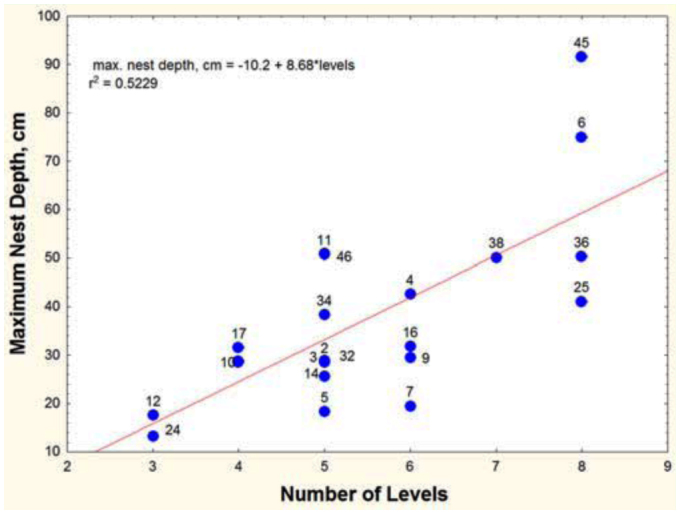
*Aphanogaster floridana.* Number of “levels” and nest depth are strongly related, but within any given number of levels, maximum nest depth can differ more than twofold because the spacing between chambers differs. High quality figures are available online.

Nests of *A. floridana* ranged from 2 to 11 chambers, from 16 to 228 cm^2^ in total area, and from 13 to 92 cm in depth. The mean depth was 41 cm, and the median 36 cm; the first quartile was at 26 cm and the third at 50 cm. The relationships between nest size variables are shown in Figures 5 and 6 and Table 1. Not surprisingly, maximum nest depth, number of chambers and mean chamber area are all strongly and positively related to total area (Table 1). In other words, nests grow by deepening, adding chambers, and enlarging chambers, but these processes contribute differently to nest growth, and these variables did not all change at the same rate. Total area increased more rapidly than did the number of chambers, so the ratio of the two (mean chamber area) increased with total area; that is, chamber size increased with nest size (Table 1, line 1). For every square centimeter increase in total area, mean chamber area increased by about 0.08 cm^2^ (Figure 5).

**Table 2.**
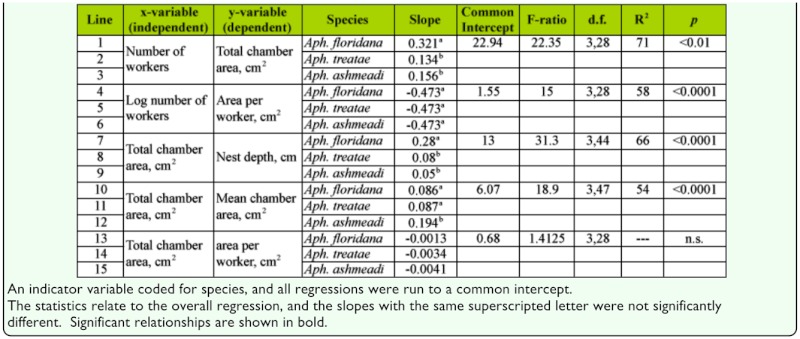
Comparison of the regressions including all three species.

Each additional chamber added an average of about 17 cm^2^ to the total area (Table 1, line 4), and each centimeter of deepening added about 2 cm^2^ (line 7). Every additional chamber was associated with a nest about 5.2 cm deeper (line 10). Mean chamber area was not significantly related either to maximum nest depth or to the number of chambers (lines 13 and 16).

More surprisingly, spacing between chambers was not significantly related to the number of chambers (Table 1, line 22). Rather, the chambers of deeper nests were spaced farther apart, a feature that can be seen in Figure 2, in which nests are grouped by “levels” (multiple chambers at about the same depth, served by different shafts, can be grouped as a “level”). Regression shows that maximum nest depth increased with the number of levels (Figure 6; maximum nest depth = -10.2 + 8.68 (levels); R^2^ = 52%), but for nests of a given number of levels (e.g., 3, 5, 6 and 8 levels), the maximum nest depth could differ by about twofold, because the spacing between levels was different. Adding more chambers (or levels) to a nest does not cause them to be spaced more closely or, for that matter, differently at all as spacing is independent of chamber number (Table 1, line 22, n.s.) but contributes significantly to maximum nest depth (line 19). For every centimeter increase in the average spacing between chambers, the nest was 4.7 cm deeper. In other words, some colonies with similar numbers of chambers space them farther apart, increasing the maximum nest depth. The reasons for these differences are unknown.

The ratio of total chamber area to maximum depth is an index of chamber area per centimeter of depth. This ratio increased with nest size (measured as total chamber area), indicating that the area available per centimeter of maximum nest depth increases with total nest size (area per centimeter depth = 1.51 + 0.0091 (total area); F_1,143_ = 51.1; p < 0.00001; R^2^ = 26%). In other words, total chamber area increases faster than nest depth.

### Number of workers and nest size, *A. floridana*

**Figure 7.**
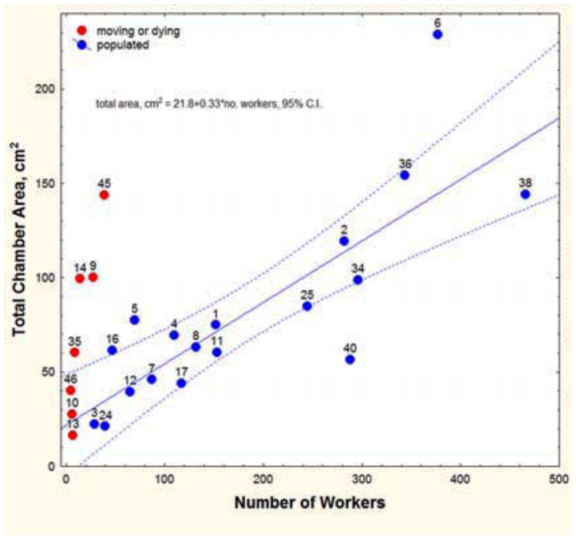
Total nest chamber area in relationship to the number of workers of *Aphaenogaster floridana.* The number next to each point refers to the nest number in Figure 2. Red symbols indicate nests that were moving or dying and were not used in the regression. High quality figures are available online.

Nest size is best understood in relationship to the ants that occupy the nests. Obviously, larger nests are generally occupied and constructed by more workers (Figures 7, 20), but an accurate assessment requires accounting for nests that are dying or moving, for the worker number to size relationship of such nests is clearly not “normal.” Nests that contained very few workers in proportion to their area (>2 cm^2^ per worker) usually also lacked a queen and were judged to be dying or moving. The seven queenless nests of *A. floridanus* averaged 22 workers, the five queenless nests of *A. treatae* 5.6, and the six queenless nests of *A. ashmeadi* 10. In the same order, nests with gynes averaged 181, 197, and 152 workers. Queenless nests were not included in regressions involving worker number below, but are shown as the red symbols in Figures 7 and 20.

For *A. floridana,* after this adjustment, every additional worker was associated with an additional 0.057 cm^2^ of floor space (Figure 7; Table 1, line 31). Although nest area increased with the worker population, it did so at a lower rate, so that workers were more crowded in large than in small nests. The area per worker declined from about 0.8 cm^2^ in small nests to about 0.2 cm^2^ in very large nests (Table 2, line 4).

Maximum nest depth was also strongly related to the number of workers in *A. floridana.* For every additional worker, the nest was about 0.09 cm deeper (Table 1, line 34). For every 100 workers, the nest had one additional chamber (Table 1, line 37).

### Size-free shape, *A. floridana*

**Figure 8.**
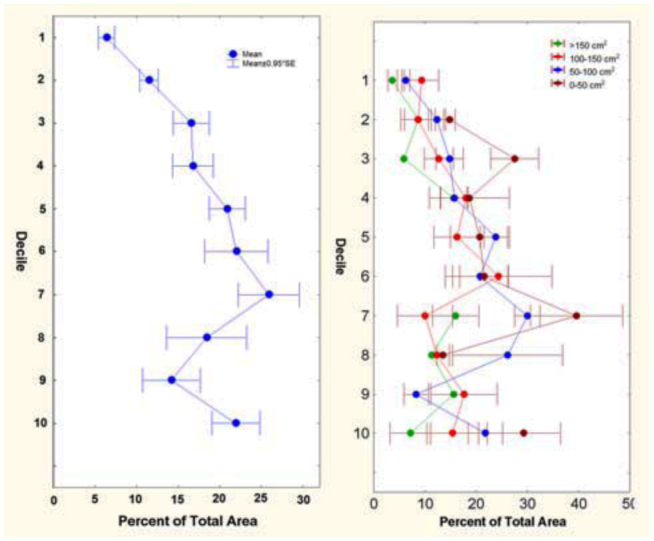
(A) Size-free nest shape. The distribution of percentage of total chamber area versus relative depth, where depth is represented in tenths of the maximum (deciles), from surface to the bottom. (B) The same relationship for four size classes of nest based on total area. The shape of the nest did not change with nest size. High quality figures are available online.

Shape of the nest was estimated independently of size (Mosimann and James 1979) from plots of the percentage of the total chamber area against decile, where decile represented tenths of the maximum depth from the surface to the bottom. Such plots showed that nests tended to be “bottom-heavy”; that is, a significantly higher proportion of the total area was in lower regions of the nest, especially in the 7^th^ and 8^th^ deciles (Figure 8A). The percentage of total area changed significantly with decile, but this relationship did not change with total colony size or interact with colony size (Figure 8B; 2-way ANOVA of log percentage total area; F_5,106_ = 2.91; p < 0.02). This pattern was very similar for mean chamber size plotted against decile; the largest chambers occurred in the 6^th^ and 7^th^ deciles. The general architecture, in the sense of the vertical distribution of chamber area, is therefore independent of scale. The ants build nests of the same “shape” no matter what the size. Therefore, no modification of the “rules of excavation” is necessary as the ants enlarge their nest.

**Figure 9.**
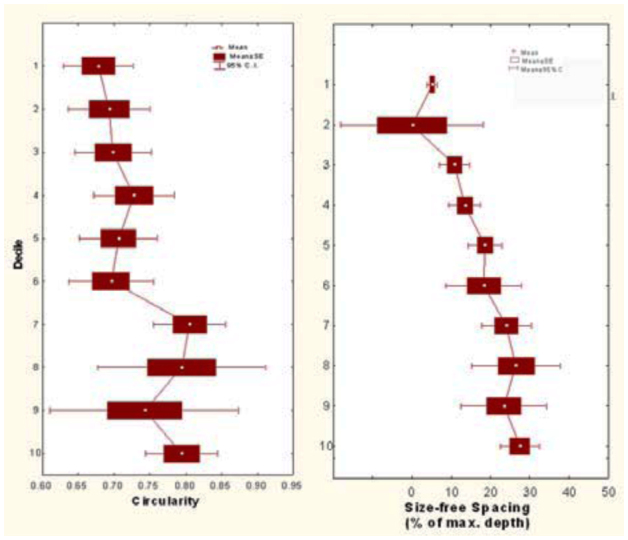
(A) Size-free nest shape. The circularity of chamber outlines was greater in the bottom half of the nest than in the top. This change in chamber shape did not depend on nest size. (B) Size-free spacing of chambers increased with decile, indicating that deeper chambers were farther apart. High quality figures are available online.

Another size-free shape question regards the horizontal outlines of chambers. Figure 9A shows the circularity of chambers (the ratio of chamber perimeter to the perimeter of a circle of the same area) in relation to the decile in which the chamber resides. Both are size-free, unitless variables. Chambers in the lower deciles are significantly more circular than those higher in the nest (Figure 9A; one-way ANOVA, F_9,132_ = 2.86; p < 0.005). A two-way ANOVA with decile and size class showed that this pattern did not depend on nest size. Because chamber size increased with decile (Figure 8A), circularity might be related to chamber size. Indeed, the regression was significant, but the explained variance was only about 3%.

The spacing between sequential chambers can also be expressed as a size-free variable computed as percentage of the maximum nest depth. This size-free spacing increased with decile (Figure 9B; one-way ANOVA, F_9,135_ = 5.75; p < 0.00001), indicating that the deeper in the nest, the farther chambers are apart, no matter what the nest size (two-way ANOVA with decile and size category, the latter n.s.). This pattern is readily recognizable in Figure 2.

### Architectural features of *A. treatae* nests

**Figure 10.**
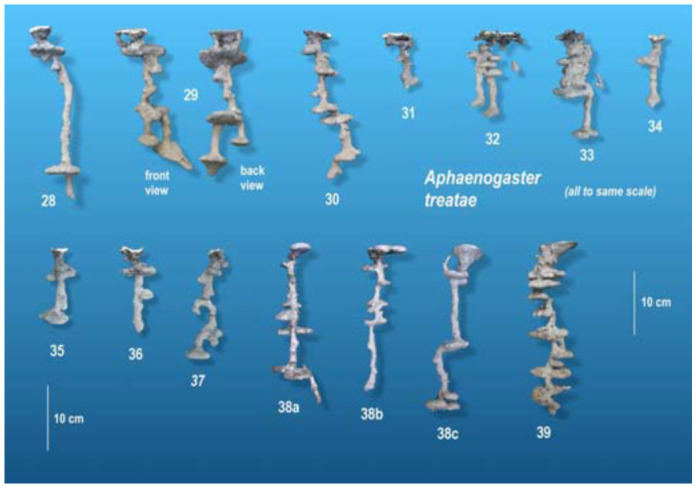
*Aphaenogaster treatae* nests, all to the same scale. The number under each cast is the number of the Appendix image in which a larger stereo image of the cast can be found. High quality figures are available online.

**Figure 11.**
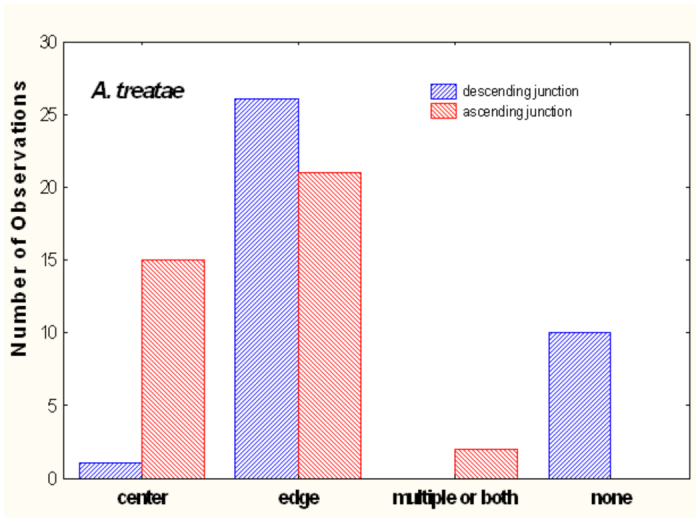
In *Aphaenogaster treatae*, shafts connecting to chambers above were more likely to arise from the center of a chamber than were descending shafts, whereas the two were about equally likely to arise from the edge. Multiple shafts connecting chambers were uncommon. High quality figures are available online.

In contrast to the crisp architectural features of *A. floridana, A. treatae* nests seem simple and somewhat sloppy (Figure 10). Most nests consisted of a single more or less vertical shaft connecting simple chambers. As in *A. floridana*, descending shafts tended to arise from the edges of chambers, whereas those connecting to chambers above tended to arise from the centers (Figure 11), but this pattern was less consistent than in *A. floridana.* The nests lacked the often multiple shafts seen in the upper regions of *A. floridana* nests, although multiple entrances from the surface were sometimes present (Figure 10, nests 29, 31). Only two nests (29, 32) had two shafts in the lower parts of the nest.

**Figure 12.**
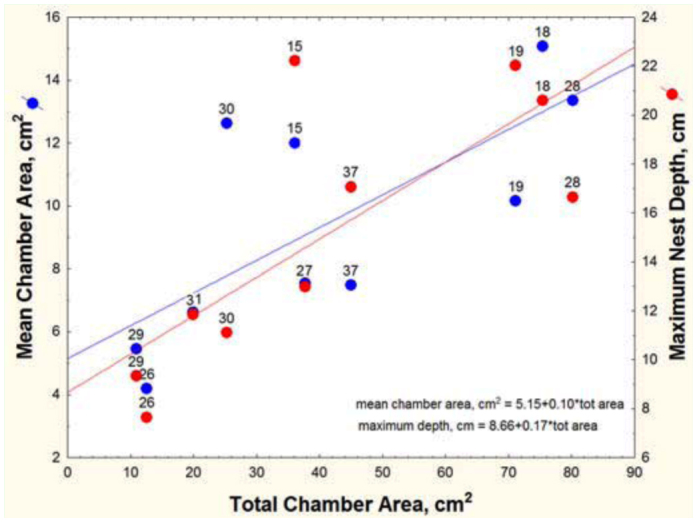
Mean chamber area and nest depth both increased with total chamber area in *A. treatae* nests; that is, nests grew by simultaneous enlargement of chambers and deepening. High quality figures are available online.

The nests ranged in size from two to seven chambers (nine, including the metal cast of nest 39), 8 to 22 cm depth, and 11 to 80 cm^2^ total chamber area. As in *A. floridana*, nests grew by simultaneously deepening, enlarging of chambers, and addition of chambers (Table 1; Figure 12). For every square centimeter of increase in mean chamber area, the total area increased by about 5 cm^2^ (Table 1, line 2), and for every centimeter of depth, total area increased by 3.8 cm^2^ (line 8). Every additional chamber increased total area by about 12 cm^2^ (line 5). Mean spacing, maximum depth, and number of chambers were not significantly related (Table 1, lines 20, 23). Using both maximum depth and number of chambers as predictors increased the explained variation to about 80% (line 26) but decreased the contribution of each chamber to 8.3 cm^2^ and rendered nest depth nonsignificant.

### Worker number and nest size in *A. treatae*

The relationship between worker number and total chamber area was not significant in *A. treatae* (Table 1, line 32), probably because of the small sample size.

### Size-free nest shape of *A. treatae* nests

**Figure 13.**
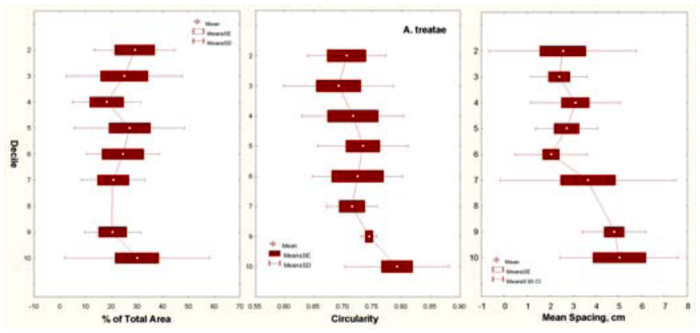
*Aphaenogaster treatae.* Three size-free measures of nest shape, in relation to depth decile. (A) Percentage of total area. (B) Chamber circularity. (C) Size-free chamber spacing. Although the right two both increased toward the bottom of the nest, this increase was not significant. Percentage of total area was evenly distributed across decile, and this distribution did not change with nest size. High quality figures are available online.

The vertical distribution of chamber area (percentage of total area) was remarkably even, showing no significant relationship to depth decile. Area was not concentrated in any vertical region of the nest (Figure 13 A; one-way ANOVA, n.s.), and this characteristic did not change with nest size. The chambers changed little in shape (as measured by circularity) as their size increased (regression, n.s.). The size-free relationship of circularity to decile revealed an increase of circularity toward the bottom of the nest, but this increase was not significant (Figure 13B; one-way ANOVA, n.s.). Similarly, the size-free mean spacing (proportion of maximum nest depth) between chambers increased from the upper parts of the nest toward the lower parts, but these differences also proved not to be significant (Figure 13C; one-way ANOVA, n.s.). The general shallowness of the nests probably precluded these kinds of differences, although a larger sample size might show them to be significant. All in all then, the size-free shape of *A. treatae* nests, by several different measures, changed little with size and showed no significant vertical pattern. A perusal of Figure 10 can convince the viewer of the validity of this conclusion.

### Architectural features of *A. ashmeadi* nests

**Figure 14.**
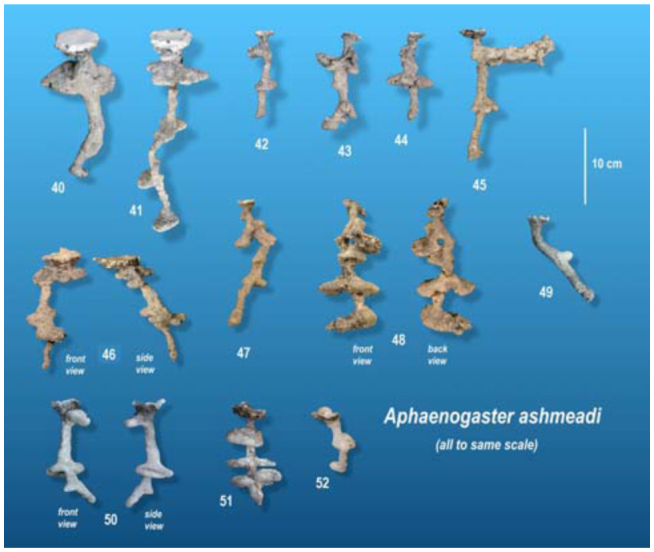
*Aphaenogaster ashmeadi* nests, all to the same scale. The number under each cast is the number of the Appendix image in which a larger stereo image of the cast can be found. High quality figures are available online.

**Figure 15.**
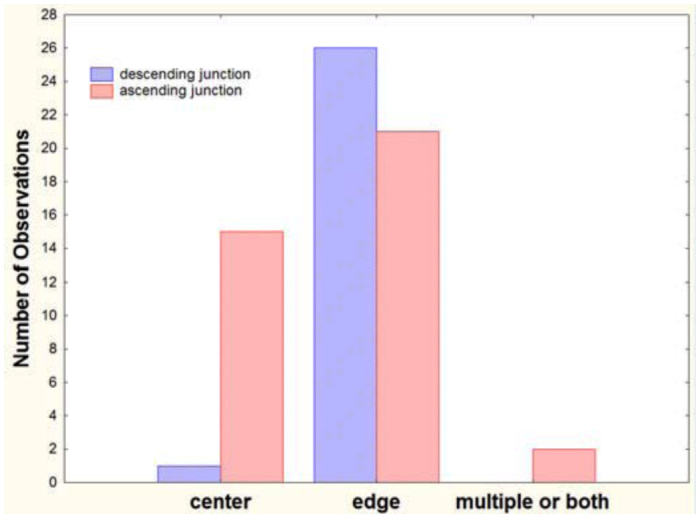
In *Aphaenogaster ashmeadi*, shafts connecting to chambers above were far more likely to arise from the center of a chamber, whereas descending shafts were equally likely to arise from the center or the edge. High quality figures are available online.

The nests of *A. ashmeadi* seem poorly defined compared to *A. treatae* nests, and especially to the neatly defined *A. floridana* nests. The chambers were mostly small and sometimes not well differentiated from the shafts, the shafts were relatively plump and were sometimes vertical and sometimes inclined (Figure 14). As in *A. floridana* and *A. treatae, A. ashmeadi* shafts connecting to chambers above tended to arise from the centers of chambers, whereas descending shafts arose about equally from the center and the edge (Figure 15). Few multiple shafts connected chambers.

**Figure 16.**
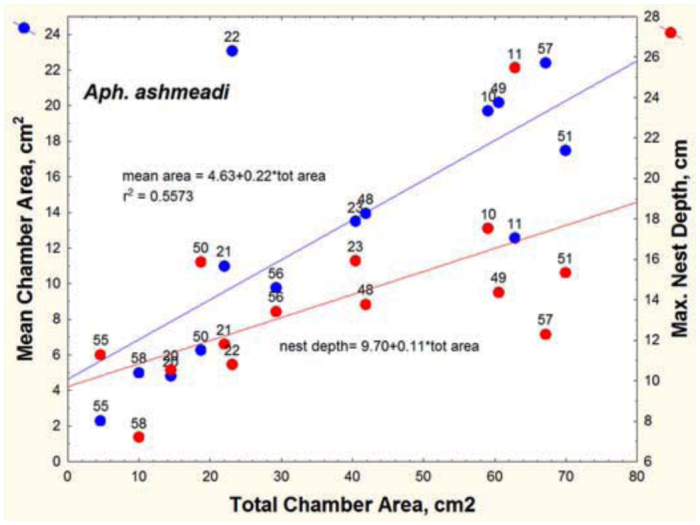
Mean chamber area and maximum nest depth of *Aphaenogaster ashmeadi* nests as a function of total chamber area. Mean area increased more rapidly than nest depth as total area increased. High quality figures are available online.

Total chamber area ranged from 10 to 69 cm^2^, depth from 7 to 25 cm, and number of chambers from 1 to 4. Of the three species, these were the smallest nests. Every square-centimeter increase in total chamber area was associated with a 0.22 cm^2^ increase in the mean chamber area (Figure 16; Table 1, line 3). Each additional chamber added about 19 cm^2^ to the total area and about 3.7 cm to nest depth (lines 6, 12). On the other hand, mean chamber area was not significantly related to nest depth (line 15), and mean spacing was related neither to nest depth nor to number of chambers (lines 21, 24). These appear to be independent elements of nest architecture. When both nest depth and number of chambers were used as independent variables, the combination was not significantly related to total area (line 27).

### Worker number and nest size in *A. ashmeadi*

**Figure 17.**
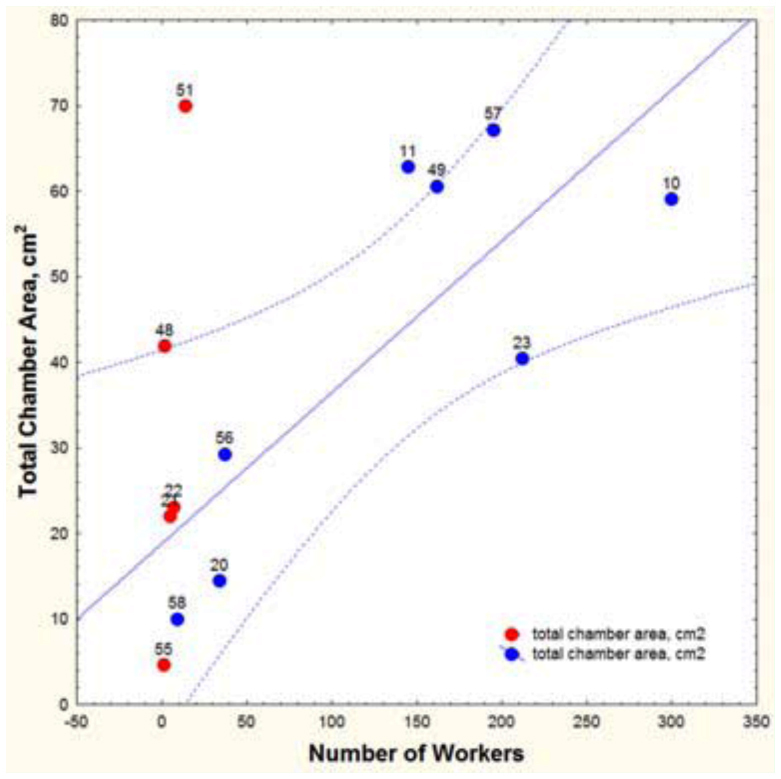
*Aphaenogaster ashmeadi.* Total chamber area in relation to the number of workers. The number next to each point refers to the nest number in Figure 15. Red symbols indicate nests were moving or dying and were not used in the regression. High quality figures are available online.

In *A. ashmeadi*, the relationship of worker number to total nest area was significant but not strong (Figure 17, Table 1, line 33). Each additional worker was associated with a mean addition of 18 mm^2^ to the nest area, but the result was greater crowding in larger nests. Small nests contained about 0.8 cm^2^ per worker and large ones 0.2 cm^2^ (log regression: Table 2, line 6). Three nests were empty or nearly so, suggesting that *A. ashmeadi* moves frequently. They were not used in the regression.

Nests of *A. ashmeadi* seemed particularly prone to abandonment. Of the 14 nests, five contained very few workers in relation to their size. In one case, workers were seen moving brood from nest 48 to the somewhat larger nest 49. Both nests were cast and processed, and the result established that nest 48 had only two workers, whereas nest 49 had 162 plus a queen.

**Table 3.**
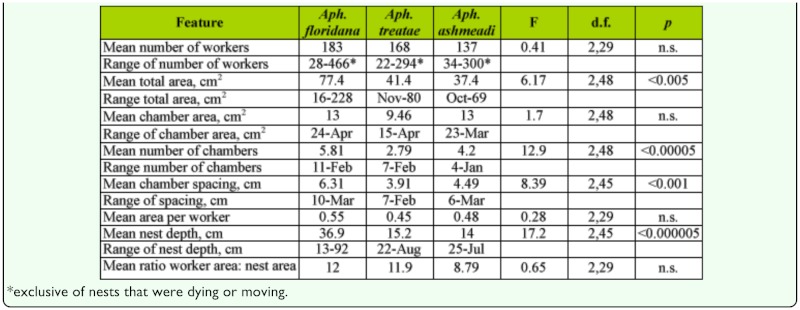
A comparison of the structural features of the three species of Aphaenogaster. Statistics are from tests of differences among the species.

### Size-free nest shape of *A. ashmeadi* nests

**Figure 18.**
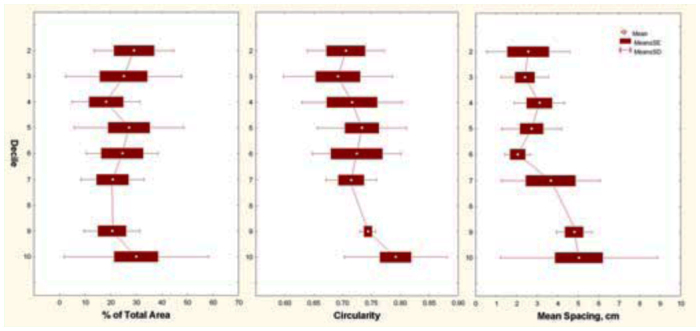
*Aphaenogaster ashmeadi.* Three size-free measures of nest shape in relation to relative nest depth (decile) indicate that chamber area, chamber shape (circularity), and spacing were all evenly vertically distributed. Moreover, this pattern did not change with nest size. High quality figures are available online.

As in *A. treatae,* nests of *A. ashmeadi* showed an even vertical distribution of chamber area (Figure 18; one-way ANOVA of percentage total area versus decile, n.s.). Similarly, although circularity and mean spacing were somewhat higher in the deeper parts of the nests, this pattern failed to approach significance (one-way ANOVA, n.s.); the architecture showed no vertical differentiation. Moreover, none of these patterns were significantly different in nests of different sizes, suggesting that shape was vertically uniform for nests of all sizes. As with *A. treatae*, this uniformity may be the result of the small nest size, and gives the measures little statistical power.

### Comparison of the three species

**Nest size variables.** The nests of the three species differed in size and in size range (Table 3). *A. floridana* had by far the largest nests, and *A. ashmeadi* the smallest; *A. treatae* was usually intermediate but closer to *A. ashmeadi* than to *A. floridana.* The largest nests of *A. floridana* were more than three times the size of the largest *A.* *ashmeadi* nests.

The three species did not differ significantly in average chamber size (Table 3; one-way ANOVA, n.s.), averaging 9.5 to 13 cm^2^, so the interspecies differences resulted from differences in the number of chambers and the depth of the nest (Table 3). *A. ashmeadi* had significantly fewer chambers than *A. treatae* or *A. floridana.*

**Figure 19.**
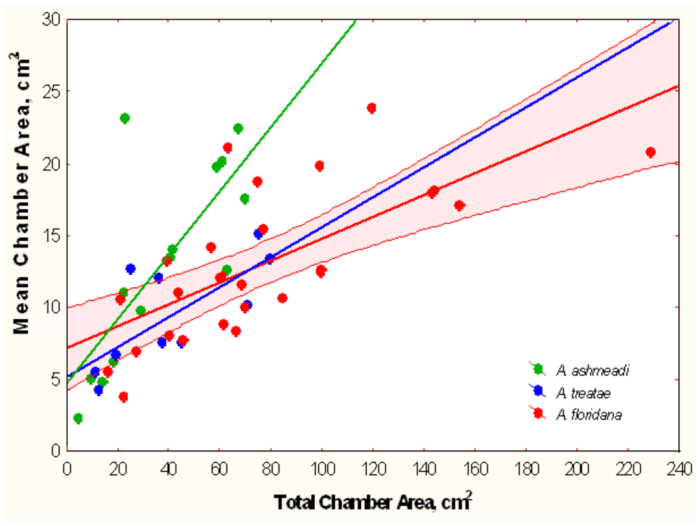
Because its nests had fewer chambers, the mean chamber area of *Aphaenogaster ashmeadi* increased more rapidly with total area than did those of the other two species. For simplicity, the 95% confidence limits are shown only for *A. floridana.* Regression statistics in Table 2, lines 10–12. High quality figures are available online.

However, mean chamber area of *A. ashmeadi* increased significantly faster with total area than did those of the other two, a probable consequence of having fewer chambers (Figure 19, Table 2, lines 10–12). For the same increase in total area, nests of *A. floridana* deepened 3 to 4 times more than did those of the other two (Table 2, lines 7–9).

**Figure 20.**
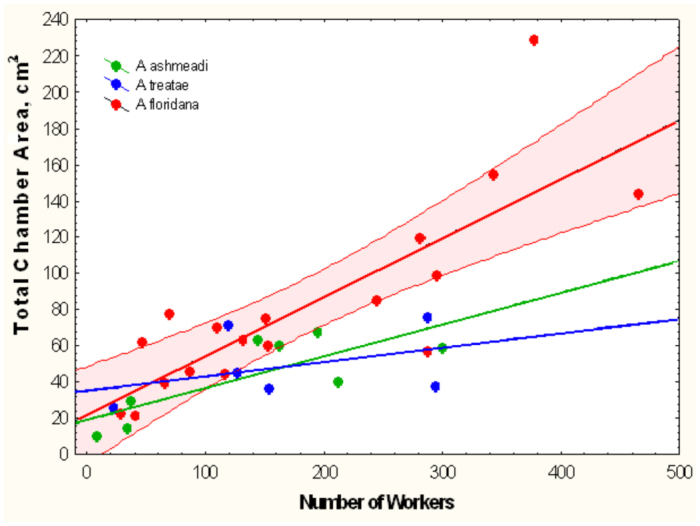
Total chamber area increased with the number of workers but did so significantly more rapidly for *Aphaenogaster floridana* than for *A.* *treatae or A. ashmeadi.* For simplicity, the 95% confidence limits are shown only for *A. floridana.* Regression statistics in Table 2, line 1–3. High quality figures are available online.

**Worker number and nest size.** The total area of nests of *A. floridana* increased significantly more rapidly with worker number than did those of *A. treatae* and *A. ashmeadi* (Figure 20, Table 2, lines 1–3), but remember that worker number was not significantly related to total area in a regression of *A. treatae* alone. Figure 20 also makes clear that the largest nests of *A.* *floridana* had almost twice as many workers as the other two species.

**Figure 21.**
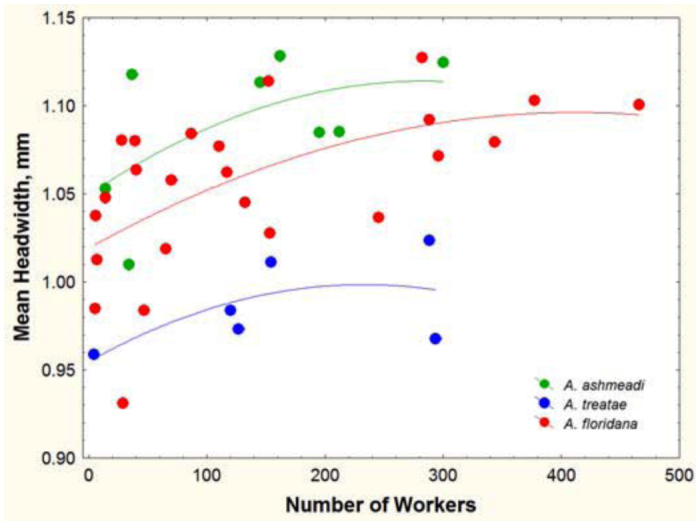
The mean head width of workers increased about 10% as colonies increased from very few workers to hundreds, but the relative sizes of workers of the three species remained the same at all colony sizes; *Aphaenogaster ashmeadi* workers were the largest and those of *A. treatae* the smallest. The curves are fitted polynomials with similar slopes but different intercepts. High quality figures are available online.

**Nest size, worker size, and crowding.** The species differed somewhat in body size. *A. ashmeadi* workers were the largest at 6.6 mm in body length, with a dorsal silhouette of 5.5 mm^2^. *A. floridana* followed at 6.2 mm in length and 4.6 mm^2^ in dorsal silhouette. *A. treatae* was 5.2 mm and 3.8 mm^2^. The dorsal silhouette probably underestimates the actual area needed by a worker ant, but estimation of the silhouette with legs is fraught with uncertainty, and is probably roughly proportional to the body-only silhouette. Body size, as estimated by head width, was itself related to the number of workers in the nest (Figure 21). Head width increased by about 10% as colonies grew from few workers to hundreds. By extension, body size increased by a similar amount. The pattern of increase was similar for all three species, and their relative size remained the same at all colony sizes.

**Figure 22.**
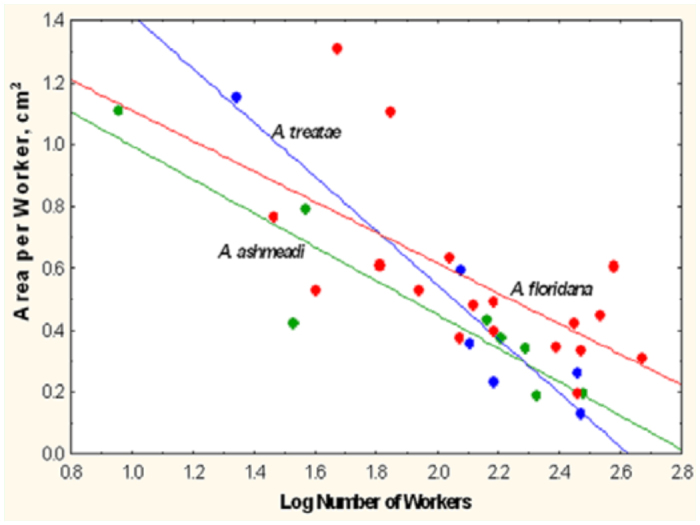
The area per worker declined as colonies increased in worker population, but each added worker had a smaller effect than the previous one (i.e., the relationship was logarithmic). The regressions for the three species were not significantly different in slope or intercept (*t*-test; Table 1, lines 4–6). High quality figures are available online.

The area per worker decreased at a similar rate with the number of workers in all three species (Figure 22; Table 2, lines 4–6), so crowding increased as the worker population increased. This decrease was logarithmic, so each additional worker was associated with a smaller decline in space per worker than the previous one (Figure 22). This result would seem to contradict the evidence in Figure 20 but is the outcome of the considerable variation around the regression line, such that nests with the same number of workers differed by several fold in area and therefore in area per worker.

Combining the area per worker with the dorsal silhouette area shows that worker bodies occupied about 3–5% of the space in nests with 10 individuals, but 23–28% in nests with 400 individuals, an increase in crowding of 5-to 6-fold. But, in general, the species were not so very different in crowding (nests that were moving or dying were not used in this calculation). When the femurs were included in the silhouette, the used space increased about 3-fold, i.e., to 12–15% in small colonies and about 70–80% in large ones. The area occupied by brood could not be determined.

**Figure 23.**
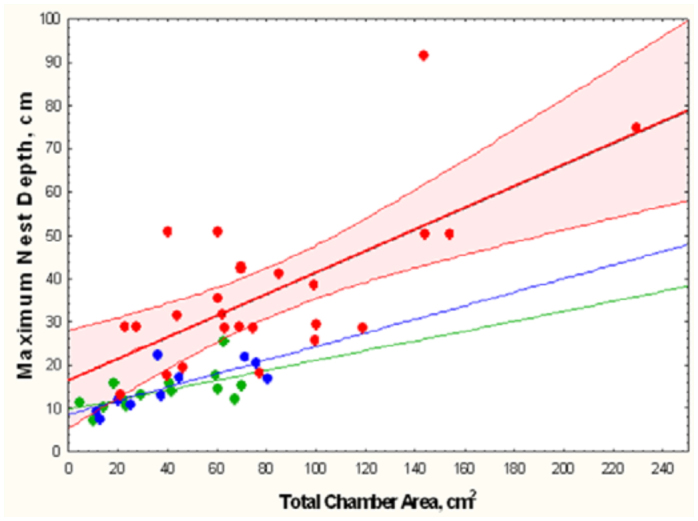
Nests of similar size were significantly deeper in *Aphaenogaster floridana* than in the other two species, and their depth increased more rapidly in relation to nest size. The differences in size range are also apparent. For simplicity, the 95% regression confidence limits are shown only for *A. floridana.* Regression statistics in Table 2, lines 7–9. High quality figures are available online.

Nests of *A. floridana* of similar areas were deeper than those of the other two species, and they increased in depth more rapidly as well (Figure 23; Table 2, lines 7–9). These allometric differences are also apparent in Figures 2, 10, and 14.

## Discussion

The architectures of the three species of *Aphaenogaster* share a number of elements. All are mostly vertical and relatively small, with vertical shafts and little horizontal spread, near-circular chambers of roughly the same size housing workers at similar densities, a chamber close to the surface, and descending shafts emanating from chamber edges and connecting to the centers of the chambers below. The nests differed in several ways as well; those of *A. floridana* were much deeper and included more chambers than those of the other two, reflecting the larger colony size of this species. Chambers in the upper parts of *A. floridana* nests were connected by multiple shafts, a feature mostly lacking in the other two species. Nests of *A. treatae* and *A. ashmeadi* lacked the crispness of structure so conspicuous in *A. floridana.* Differences in nest size were associated only with an increase in the number of chambers (which in turn were associated with deeper nests) and not in mean chamber size.

The “shape“ (proportions) of the nests of all three species was independent of scale, that is, similar at all sizes of nest. To a large extent, this characteristic is what gives the architecture its species-typical appearance, as it does also in nests of *Odontomachus brunneus* (Cerquera and Tschinkel 2010), *Pogonomyrmex badius*, and *Camponotus socius* (Tschinkel 2004, 2005). A size-independent shape also means that workers can use the same excavation algorithm throughout nest growth, simplifying the evolution of the behavioral programs involved in nest construction. Vertical shafts connecting horizontal chambers are a widespread architectural unit among subterranean ant nests. This suggests that the ancestors of the ants seem likely to have dug such burrows, though probably with a single or very few chambers.

### Crowding, nest size and worker number

The total chamber area of most ant nests is probably proportional to the number of ants in the nest because presumably the ants construct the nest as living space in some proportion to their needs. The degree of crowding and the variation of crowding among species and across nest sizes could be important traits that evolved in response to some colony function. Because ants range enormously in body size, from tiny thief ants to colossal *Paraponera*, a direct comparison of architecture across species cannot easily be made, but such comparisons can be made by means of measures of crowding that are size-free, both with respect to the body size of the ants and the size of their nests. One such size-free measure of crowding is the proportion of the nest's total area that is taken up by the bodies of the ants. This crowding index is the ratio of the mean area of the worker dorsal silhouette (measured from digital photos) to the area per worker, expressed as a percentage (Table 4). The measures from the data for *Pogonomyrmex badius* (Tschinkel, 2004), *Camponotus socius* (Tschinkel 2005), and *Odontomachus brunneus* (Cerquera and Tschinkel 2010) were computed in order to compare them with those of the three species of *Aphaenogaster.* For the polymorphic *C. socius*, this calculation took into account the actual distribution of worker sizes. (An unknown ingredient in crowding is whether the ants use the ceilings of their chambers as well as the floors. If they did, the available area would almost double. They probably do not, for in laboratory nests, the great majority of the ants remain on the floor).

**Table 4.**
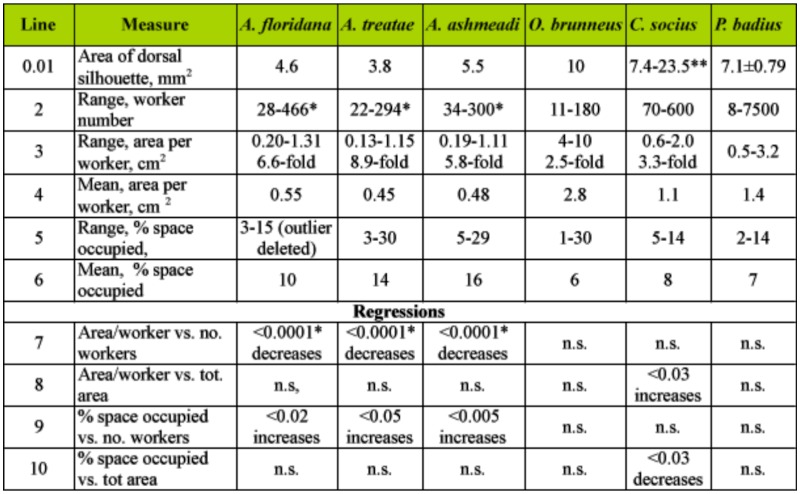
Comparison of nest occupancy in 6 species of ants.

In *Aphaenogaster*, the size-free crowding index (percentage of space occupied by worker silhouette) increased significantly from around 4% up to about 25% as the worker population increased from 10 to about 400; that is, crowding increased 5- to 7-fold. If legs were included in the silhouette, these figures were about 14 to 75%. In contrast, in no other species was this index of crowding related to the size of the worker population (Table 4). For example, *C. socius* workers consistently occupied about 8% (SD 2.6%; n = 12) of total space, *O. brunneus* about 6%, and *P. badius* about 7% as colonies grew by manyfold in the number of workers (regression, n.s.). With the exception of *C. socius*, the same was true in the relationship of percentage space occupied to total nest area (Table 4). *C. socius* became less crowded as nest area increased almost 10-fold (regression: percentage of area occupied = 11.5-0.014 (total area); F_1,10_ = 6.9; R^2^ = 35%; p < 0.03). In the smallest nests, ant bodies occupied about 13% of the area, but this figure decreased to about 5% in the largest. Including legs, these values would be approximately 2.5-fold larger. In contrast to *Aphaenogaster, C. socius* nests became less crowded as nest area increased but did not change density as the worker population increased.

Also noteworthy is that, in addition to the increase in crowding with worker number, the species of *Aphaenogaster* were more crowded on average than the other three species; their means were 10 to 16%, contrasting with 6 to 8% for the three unrelated species.

To some degree, the creation and use of space is mysterious, for most ant species do not space themselves out evenly in the available area, that is, they do not seek to minimize crowding. Rather, they crowd into a few chambers at very high density. For example, *P. badius* worker and brood density was consistently much higher in the lowest chambers of the nest, and lowest in the upper, even though the lowest chambers tended to have the least room and the uppermost the most (Tschinkel 1999). This was also the case for A. *floridana, Prenolepis imparts,* and *C. socius* but is less obvious in A. *ashmeadi* and *A. treatae* (personal observations). In *O. brunneus*, the ants are vertically evenly distributed (Cerquera and Tschinkel 2010).

The structure of *Aphaenogaster* nests suggests that the ants follow a protocol during construction. First, shafts were almost always vertical, suggesting orientation to gravity (downward). New shafts are initiated at chamber edges, possibly when crowding at the edges reaches a threshold. When excavating at greater depths, workers of *A. floridana* dig either faster or longer (or both), resulting in greater spacing between chambers. This part of the protocol is weaker in the other two species, but their nests are also shallower, possibly reducing the stimulating effect of depth. In all species, once the workers have stopped deepening the shaft, they dig radially and equally outward to create a more or less circular chamber with the ascending shaft at its center. In *A. floridana*, the stimulus to dig new shafts to greater depth was so strong in the upper reaches of the nest that multiple shafts connect the upper chambers, mostly emanating from the edges.

Filling subterranean ant nests with a casting material can provide more information than just the nest's architecture, it can also be used to determine the distribution of workers within the vertical nest structure (Cerquera and Tschinkel 2010; Tschinkel 2010). By using paraffin wax to make nest casts, the workers, brood, and alates are fixed at their momentary locations within their ant nests (unpublished data). Melting these casts in sections provides an accurate picture of the distribution of all colony members, brood, and food within the vertical nest structure. The recovered ants can also be used for other studies, such as morphometry. Compared to a simple excavation, such casting methods offer the advantage that the casting material finds and fills all the nooks, crannies and cavities of the nest, capturing all the nest contents in place, something that is difficult to achieve during direct excavation of an uncast nest.

The connection between nest architecture and colony function has received little attention, in part because most studies have been carried out in single-chambered laboratory nests that do not resemble the natural nest. Brian (1956) showed that ants in smaller groups rear brood more efficiently than those in larger groups, a result confirmed by Porter and Tschinkel (1985). Nest architecture combines with the tendency of all ants to sort themselves and their brood to produce social structure within the nest. In most species, as workers age, they move centrifugally away from the brood (Hölldobler and Wilson 1990; Sendova Franks and Franks 1995), a movement that is connected to age polyethism. In deep, vertical nests such as those of the Florida harvester ant, *Pogonomyrmex badius*, and the winter-active ant, *Prenolepis imparts*, this movement sorts workers by age such that the youngest are located mostly in the bottom third of the nest and the oldest (defenders and foragers) near and on the surface (Tschinkel 1987, 1999). The western American harvester ants *P. subnitidus and P. rugosus* are also stratified vertically by age, with associated differences in fat content and metabolic rate (MacKay 1983). Because of the near universality of the centrifugal movement of aging workers away from the brood pile, nest architecture and spatial social structure are probably functional and contribute to colony fitness. Determining whether these links exist and how they function should be a central question in the study of ant nest architecture.

Finally, reviewers have pointed out that architectural information on a number of species of ants is now available and that some synthesis would be in order. From published papers and my additional casts of 20 ant species (unpublished observations), I can offer several generalizations. (1) Ant nests are composed of two basic modules, chambers and shafts (and possibly horizontal, narrow, near-surface tunnels in some species); (2) these modules are combined into the basic nest unit, the chamber-and-shaft, or shish-kebob, unit; (3) variation of the size and shape of the modules and of their manner of combination, produce the observed differences in architectures among species; (4) some species combine multiple shish-kebob units into a single nest, with differences resulting from the number, size, and proximity of the units; (5) general nest shape does not change during enlargement; (6) nest size is related to the number of ants, but species differ in the nature of this relationship; (7) the ants are not evenly distributed within the nest but tend to be denser toward the bottom.

## Figures and Tables

**Appendix A.**
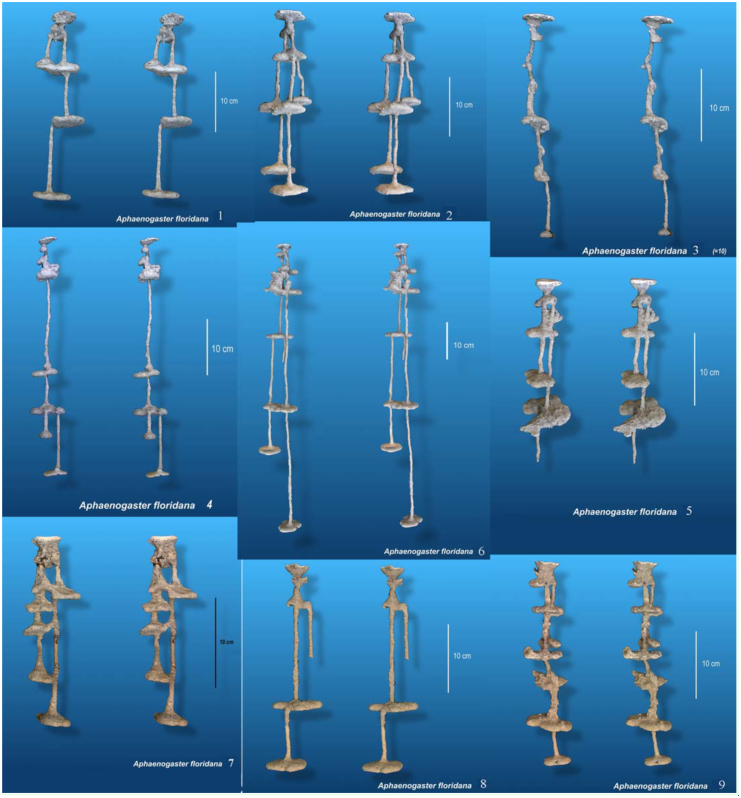
Stereo images of nest of *Aphaenogaster floridana:* images 1–27. See Figure 2.

**Appendix B.**
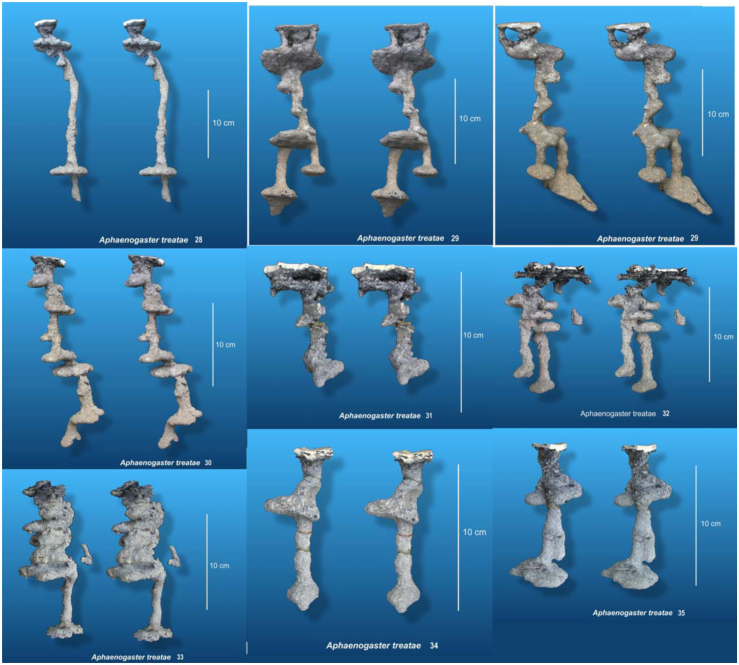
Stereo images of nests of *Aphaenogaster treatae*: images 28–39. See Figure 10.

**Appendix C.**
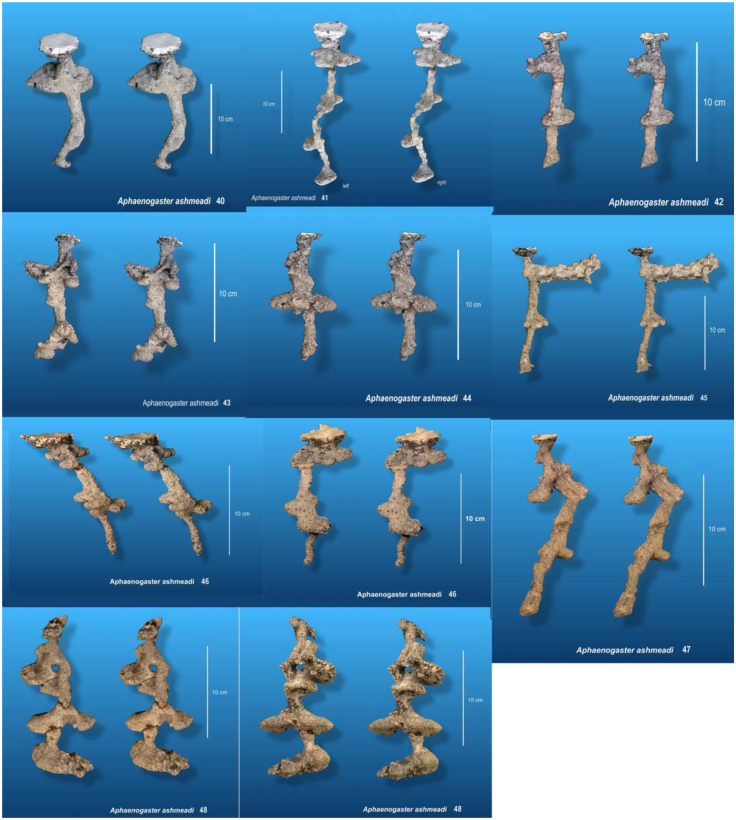
Stereo images of nests of *Aphaenogaster ashmeadi:* images 40–52. See Figure 14.
